# Solar-Driven Paired Electrolysis System: A Green Electrosynthesis Strategy for Valorizing Agroforestry Biomass Derived Furanal Compounds

**DOI:** 10.3390/molecules31040678

**Published:** 2026-02-15

**Authors:** Yi Wu, Run Xu, Bowei Wang, Changxia Sun, Xueyong Ren, Qiang Li

**Affiliations:** 1 College of Food Science, Shanxi Normal University, Taiyuan 030031, China; 2 College of Science, Beijing Forestry University, Beijing 100083, China; 3 Sinopec Research Institute of Petroleum Processing Co., Ltd., Beijing 100083, China; 4 College of Material Science and Technology, Beijing Forestry University, Beijing 100083, China

**Keywords:** biomass derived furanal compounds, paired electrolysis system, 4-acetamido-TEMPO, solar-driven

## Abstract

Paired electrolysis represents a more environmentally sustainable and efficient approach for converting agroforestry biomass-derived 5-hydroxymethylfurfural (HMF) and furfural (FUR) into valuable fine chemicals and fuel additives. A critical challenge in developing paired electrolysis systems for furanal compounds is finding the optimal potential matching between the anode and the cathode. One solution is to reduce the potential sensitivity of the anode so that the paired electrolysis system can be regulated only by the cathode potential. In this study, we employed the homogeneous catalyst 4-acetamido-TEMPO (ACT) to facilitate oxidation reaction at the anode, enabling the potential sensitivity of the anode to be reduced. The results displayed the furanal substrates oxidation proceeds through a non-electrochemical chemical reaction with the active oxoammonium cation (ACT^+^), rather than being directly governed by the anode potential. The paired electrolysis system exhibited enhanced catalytic performance, with a total faradaic efficiency of 190.69% and 189.11% in the FUR and HMF paired electrolysis setup, respectively. Furthermore, this system demonstrated excellent stability, maintaining a total faradaic efficiency of over 167.64% after multiple successive cycles. Additionally, the solar-driven paired electrolysis system showed commendable substrate conversion capabilities, achieving a total faradaic efficiency of 187.89%, comparable to that of the electrically driven system. The mechanisms of the ACT electro-oxidation of furanal compounds and the construction of paired electrolysis systems for furanal compounds were proposed and discussed. This work aims to enhance electrical energy efficiency and underscore the potential of paired electrochemical catalysis for sustainable biomass conversion in the green economy.

## 1. Introduction

Agroforestry biomass constitutes substantial renewable resources within forest ecosystems, and using it as a substitute for fossil-based feedstocks represents a viable strategy to alleviate the scarcity of energy resources [1,2]. Notably, furfural (FUR) and 5-hydroxymethylfurfural (HMF) are recognized as prominent and promising agroforestry biomass, playing a crucial role in industrial production [3,4]. Biomass-derived FUR and HMF can be converted into value-added products, including food additives, biofuels, and pharmaceutical intermediates via multiple routes [5,6]. Consequently, the transformation of agroforestry biomass-derived FUR and HMF has spurred growing interest in recent years [7,8,9,10].

The value-added conversion of biomass-derived FUR and HMF is mainly carried out through electrocatalytic reactions. This process is efficient and green, particularly when powered by renewable energy sources, such as wind or solar power, offering a waste-free alternative approach [11,12,13]. Xie et al. used a hollow CoP electrode for the electrocatalytic oxidation of HMF to 2,5-furandicarboxylic acid and achieved the 97.5% faradaic efficiency [14]. Zhu et al. achieved highly selective electro-hydrogenation of HMF to 2,5-bis(hydroxymethyl)furan and obtained the 95.7% HMF conversion [15]. Nevertheless, current studies typically focus on the conversion of furanal compounds at a single anode or cathode. The oxygen evolution reaction (OER) or hydrogen evolution reaction (HER) on the other side is neglected owing to high overpotentials, sluggish kinetics, and difficulties in gas collection, resulting in low efficiency of electrical energy utilization [16,17,18]. The simultaneous conversion of furanal compounds at both the anode and the cathode through a paired electrolysis system is supposed to overcome the problem of energy utilization. A single reaction by the paired electrolysis system can produce two kinds of valuable products, achieving a dual improvement in faradaic efficiency (theoretically up to 200.00%) and economic benefits [16,19,20].

However, the construction of paired electrolysis systems requires meeting conditions such as electrolyte compatibility and potential matching [21,22]. For the electrolyte compatibility, our previous reports have demonstrated that both electro-oxidation and electro-hydrogenation of furanal compounds can occur under mildly basic conditions, which makes it possible for the construction of paired electrolysis systems [10]. As for the potential matching, paired electrolysis systems cannot independently control the working potentials of the anode and cathode via the external circuit [22,23]. Therefore, the core issue in constructing a paired electrolysis system in this study is to solve the problem of potential matching between the anode and cathode. Considering that the efficiency and selectivity of the electro-hydrogenation are highly sensitive to the cathode potential, it is essential to find a method that can reduce the potential sensitivity of the anode, so that the paired electrolysis system can be regulated only by the cathode potential. The use of a homogeneous electrocatalyst, such as TEMPO and its derivatives 4-acetamido-TEMPO (ACT), is a promising approach to reduce the potential requirements for the anode by indirect electrocatalytic oxidation of furanal compounds [22,24,25]. Particularly, ACT with higher activity and lower cost than TEMPO is deemed as a prospective homogeneous electrocatalyst for the oxidation of furanal compounds in paired electrolysis systems [26].

Herein, we systematically explored the paired electrolysis systems of furanal compounds, which aimed to identify the optimal anodic-cathodic coupling efficiency. Furthermore, the feasibility of replacing conventional electric energy with solar energy as a green power source for the paired electrolysis systems was demonstrated. This study not only enhances the overall energy efficiency of furanal compound upgrading but also underscores the potential of paired electrocatalysis in advancing sustainable chemical conversion.

## 2. Results and Discussion

### 2.1. ACT-Mediated Electrochemical Oxidation of FUR

A homogeneous catalyst ACT was used for electrocatalytic oxidation of FUR to reduce anode potential sensitivity, so that the potential of the paired electrolysis system is controlled only by the cathode. Considering that the ACT cannot meet the demand for electron transfer in ACT-mediated FUR oxidation, it is necessary to use the electrode as the medium of electron transfer. Carbon fiber paper (CFP)and carbon cloth (CC) were used as the anode, and the optimal electron transport medium was selected by comparative analysis of their morphology and electrochemical properties. As displayed in SEM images (Figure S1), CFP is composed of smooth carbon fibers with a disordered network structure, while CC is made up of countless carbon fibers crisscrossed and woven.

The electrochemical properties of two kinds of electrodes were characterized and compared. The CV tests were performed at various scan rates within the non-Faradaic region to determine the double-layer capacitance (C_dl_) (Figure S2a,b), which is directly related to the ECSA. The linear relationships of the scan rate and Δj (Figure S2c) showed that the slope of the CC electrode possessed observably higher capacitance (1.31 mF/cm^2^) than the CFP electrode (0.91 mF/cm^2^). It is indicated that the CC electrode braided with vertically and horizontally carbon fibers has formed a three-dimensional electron transport network with a larger electrochemically active surface area and more active sites. This three-dimensional network facilitates electron transfer and benefits from providing effective current density for oxidation reactions. The result of the LSV curve was also consistent with the above results. As observed in Figure S2d, the current density of the CC electrode was always dramatically higher than that of the CFP electrode in the potential range of 0.90~2.00 V vs. RHE, indicating that the CC electrode has better electrochemical activity than the CFP electrode. Moreover, the CFP electrode exhibits brittleness and is prone to damage, whereas the CC electrode demonstrates excellent flexibility and can be folded as desired. Therefore, the CC electrode was more suitable as the medium for electron transfer in subsequent experiments.

**Figure 1:**
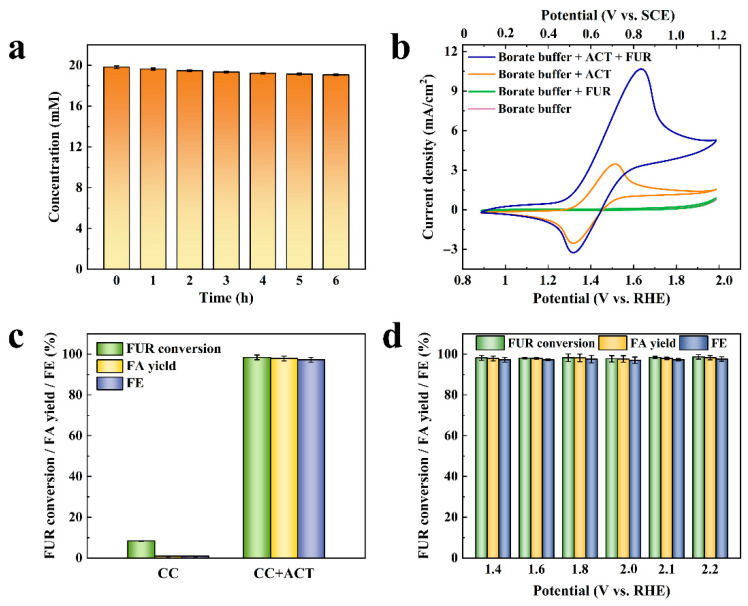
(**a**) Concentration change of FUR in ACT solution over time without applying potential; (**b**) CV curves in electrolyte with or without ACT or FUR; (**c**) Effect of ACT on the catalytic oxidation of FUR; (**d**) Catalytic oxidation effect of ACT on FUR at different potentials.

The change in FUR concentration over time was investigated in the electrolyte containing ACT and 20.00 mM FUR without applying potentials. As displayed in Figure 1a, the FUR concentration remained above 19.00 mM for 6 h with no obvious change. Meanwhile, no oxidation products of FUR were detected, indicating that ACT had no catalytic oxidation ability for FUR without applying potentials.

In order to investigate whether ACT can be used as a homogeneous catalyst for the electrocatalytic oxidation of FUR, a cyclic voltammogram (CV) was conducted. As displayed in Figure 1b, when the electrolyte contained FUR and was without ACT, the CV curves before and after substrate addition almost overlapped, and no obvious oxidation peak was observed. It is suggested that the CC electrode had no obvious electrocatalytic activity for FUR, and that non-mediated FUR oxidation could not proceed under these conditions. When the electrolyte contained ACT and was without FUR, reversible one-electron redox peaks appeared, indicating that ACT was oxidized to an oxoammonium cation (ACT^+^) during the anodic sweep and then reduced back to ACT during the cathodic sweep. When the electrolyte contained ACT and FUR, the anode peak was increased significantly, which is ascribed to the fact that ACT^+^, generated by the electrochemical oxidation of ACT, promoted the oxidation of the substrate FUR, and ACT^+^ was reduced and regenerated to ACT to participate in the subsequent oxidation reaction. It is worth noting that the cathode peak was significantly reduced compared with the anode peak, owing to the fact that only a part of ACT^+^ was consumed by FUR oxidation, while the remaining ACT^+^ was electrochemically reduced. The above result confirmed that ACT has electrocatalytic activity for FUR.

To further demonstrate the electrocatalytic oxidation of FUR by ACT, ACT-mediated FUR oxidation (30.00 mL, 20.00 mM) was carried out using a H-cell three-electrode at 2.10 V vs. RHE (1.30 V vs. SCE) with and without ACT. When the electrolyte did not contain the ACT catalyst, the FUR conversion was less than 10.00% after the electrolysis reached the theoretical conversion charge (115.00 C), and the FA yield and faradaic efficiency were also less than 1.00% (Figure 1c). This result manifested that the CC electrode had little effect on the electrocatalytic oxidation of FUR in the absence of the ACT homogeneous catalyst. When 10.00 mM ACT was added to the electrolyte, the FUR conversion was increased dramatically to 98.41%, and the FA yield and faradaic efficiency were markedly increased to 97.87% and 97.23%, respectively. This finding showed that FUR could be effectively oxidized to FA using a CC electrode as an electron transport medium and ACT as a homogeneous catalyst, and indicated that FUR was oxidized by ACT^+^ to a non-electrochemical reaction behavior [27].

Moreover, in order to investigate whether the catalytic effect of ACT is controlled by applied potentials, ACT-mediated FUR electrocatalytic oxidation tests were carried out at different potentials. As indicated in Figure 1d, the FUR conversion was maintained at about 98.00%, and the FA yield and faradaic efficiency were maintained at about 97% in the potential range of 1.40~2.20 V vs. RHE. These results indicated no obvious correlation between the applied potential and catalytic effect. This potential-independent behavior confirmed that FUR oxidation proceeded primarily through a non-electrochemical chemical reaction with ACT^+^ in solution, rather than being directly governed by the anode potential. The result suggested that it is feasible to pair FUR oxidation with FUR hydrogenation to construct an efficient paired electrolysis system.

**Figure 2:**
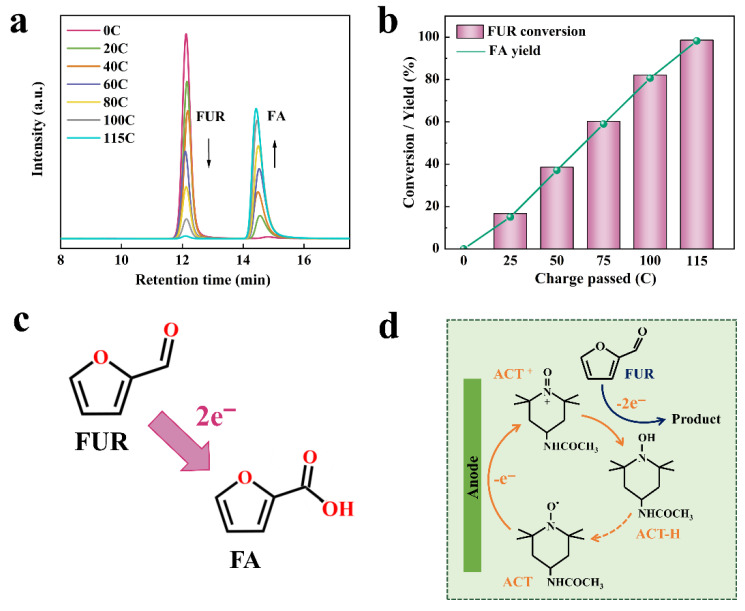
(**a**) HPLC of electrolyte components during electrolysis of FUR; (**b**) FUR conversion and FA yield during electrolysis; (**c**) Electrochemical pathway for oxidation of FUR to FA; (**d**) The mechanism pathway of electrocatalytic oxidation of FUR by ACT.

Meanwhile, the products collected from the anode cell were regularly monitored and quantified using HPLC after every 20.00 C of charge passed through in the ACT-mediated FUR electrooxidation reaction (Figure 2a). The changes in the concentrations of FUR and FA during the electrooxidation reaction were collected (Figure 2b). The results showed that FUR concentration decreased as the charge increased, while the FA concentration increased correspondingly. It is suggested that FUR was effectively oxidized to FA without other byproducts. The mechanism of ACT-mediated FUR electrooxidation is depicted in Figure 2c,d. Initially, ACT was oxidized to the active species ACT^+^. ACT^+^ had strong catalytic activity and oxidized the substrate FUR during the reduction to ACT hydroxylamine (ACT-H). Due to the electron-inducible effect of the amide group in ACT, ACT^+^ was more nucleophilic and had a greater driving force for nucleophilic attack on the substrate [28,29,30].

Theoretically, ACT regeneration has two pathways: (i) one of which is the reoxidation of the ACT-H, and (ii) the other is the comproportionation reaction between ACT^+^ and ACT-H. When the input of ACT is less than that of the substrate, pathway (i) is the main way for ACT regeneration (Figure 2d). That is, when ACT^+^ is less than ACT-H on the electrode surface, only a small amount of ACT^+^ is mixed with ACT-H, and the limited reaction rate facilitates the direct oxidation of ACT-H to ACT. When the input of ACT is greater than or equal to the input of substrate, pathway (ii) is the main way of ACT regeneration. In other words, when the concentration of ACT^+^ on the electrode surface is higher than ACT-H, the ACT^+^ will mix with the ACT-H generated after oxidation of the substrate to generate ACT [31,32]. In this study, the input of ACT (10.00 mM) was lower than that of the substrate FUR (20.00 mM), and pathway (i) was the main way for ACT regeneration.

### 2.2. Paired FUR Hydrogenation and Oxidation

**Figure 3:**
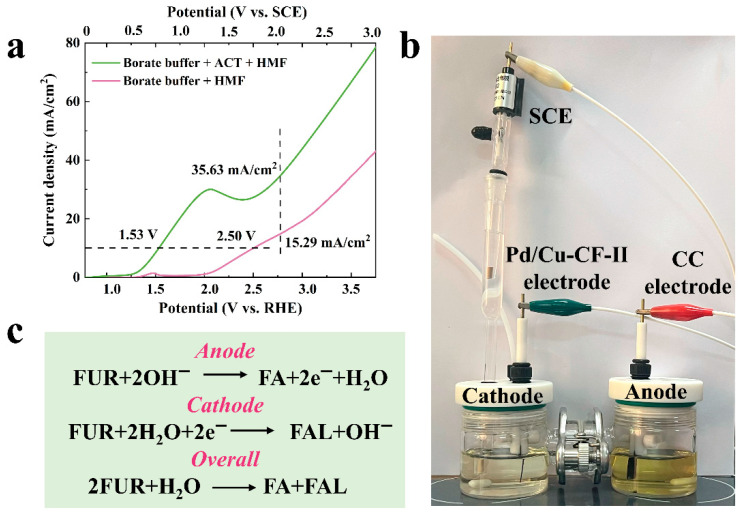
(**a**) LSV curves of FUR paired electrolysis reactions; (**b**) The device of the FUR paired electrolysis; (**c**) The anodic, cathodic and total reactions of FUR paired electrolysis.

Based on confirming that ACT-mediated FUR electrocatalytic oxidation could be carried out, the possibility of constructing a FUR oxidation-hydrogenation paired electrolysis system was further explored. As shown in Figure 3a, compared to the LSV curve without substrate FUR, the potentials at 10.00 mA/cm^2^ decreased from 2.50 V vs. RHE to 1.53 V vs. RHE, and the current density at 2.80 V vs. RHE increased from 15.29 mA/cm^2^ to 35.63 mA/cm^2^ after the addition of FUR, indicating that paired electrochemical conversion of FUR occurred more favorably and preferentially than the water electrolysis reaction.

The device of the FUR paired electrolysis is indicated in Figure 3b. Since two electrons were required to convert FUR to FA and FAL, the electrolytes in the anode and the cathode chambers were 0.5 M borate buffer solution containing 20.00 mM FUR (pH = 9.2) to ensure electron equilibrium. The anodic FUR oxidation mediated by ACT effectively acts as a counter reaction, since its potential remained unaffected. Our previous studies showed that the Pd/Cu-CF-II electrode holds better electrocatalytic hydrogenation performance for aldehyde compounds, and it was used for the electro-hydrogenation of FUR in this study [33]. The results of the Pd/Cu-CF-II electrode catalyzing FUR under different potentials were displayed in Figure S3, and indicated that the Pd/Cu-CF-II electrode exhibited the optimal electrocatalytic hydrogenation effect on FUR at −0.51 V vs. RHE. The cathode potential was controlled to an optimal −0.51 V vs. RHE (−1.30 V vs. SCE) to realize the paired electrolysis of FUR. The anodic, cathodic and total reactions of FUR paired electrolysis were displayed in Figure 3c.

**Figure 4:**
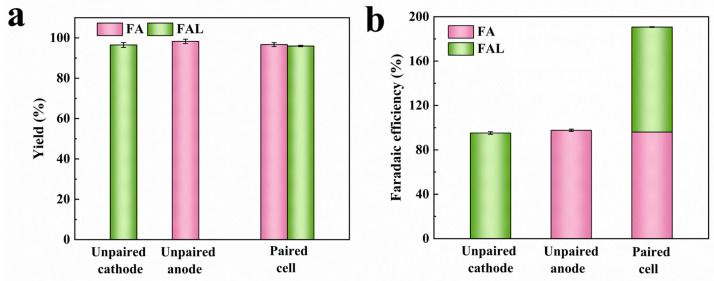
(**a**) Yield and (**b**) faradaic efficiency of FA and FAL by unpaired and paired electrolysis of FUR.

Theoretically, a charge of 115.00 C is needed to completely transform 20.00 mM FUR into either FA or FAL. Figure 4a indicates that after passing a charge of 115.00 C in the paired electrolysis system, the yields for FA and FAL achieved 96.67% and 95.96%, respectively. The yields were slightly lower than those achieved in unpaired cells (FA-98.27%, FAL-96.49%). This attenuation might be attributed to the presence of system resistance that impaired electron transport. Meanwhile, the faradaic efficiency of the FA and FAL was 190.69% in the paired electrolysis system, which was almost twice that of the unpaired reaction (Figure 4b). The findings suggested that the FUR electro-oxidation and electro-hydrogenation reactions were compatible and progressed without serious complications or adverse reactions when paired in a single cell. Each transferred electron contributed to the formation of two desired products, greatly improving the efficiency of power utilization.

### 2.3. Paired HMF Hydrogenation and Oxidation

**Figure 5:**
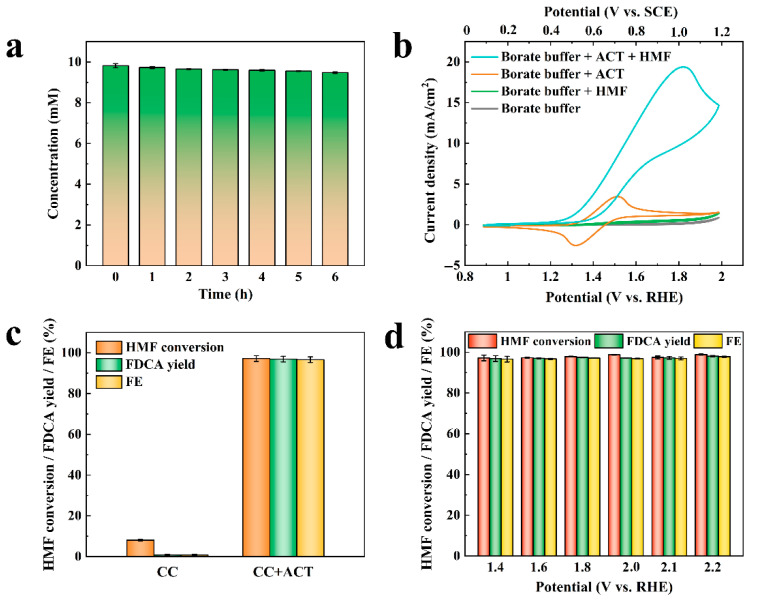
(**a**) Concentration change of HMF in ACT solution over time without applying potential; (**b**) CV curves in electrolyte with or without ACT or HMF; (**c**) Effect of ACT on the catalytic oxidation of HMF; (**d**) Catalytic oxidation effect of ACT on HMF at different potentials.

Since HMF is a derivative of FUR with a similar structure, the ACT-mediated electrochemical oxidation and paired electrolysis of HMF were also investigated. The change in HMF concentration with time in the ACT solution was investigated without applying potentials. As shown in Figure 5a, the concentration of HMF remained above 9.50 mM for 6 h without significant change, while HPLC did not detect the oxidation products of HMF. This finding suggested that the ACT-mediated catalytic oxidation of HMF could not be completed without applied potentials.

The electrochemical catalytic activity of ACT on HMF was investigated by CV. As depicted in Figure 5b, the CV curve did not change significantly before and after adding the substrate HMF, and no obvious oxidation peak was observed, indicating that the CC electrode had no obvious electrocatalytic activity for HMF. When the substrate solution contained both ACT and HMF, the anode current increased dramatically, indicating that ACT^+^ generated by the electrochemical oxidation of ACT promoted the oxidation of substrate HMF, and it regenerated into ACT to participate in the subsequent oxidation reaction. It is worth noting that the cathodic peak disappeared in the presence of HMF, which was different from the decreased cathodic peak in the presence of FUR. The reason for this phenomenon might be that the oxidation of HMF to the target substrate FDCA required six electrons, while the oxidation of FUR to the target substrate FA required only two electrons. FUR oxidation consumed part of the ACT^+^, while the remaining ACT^+^ was electrocatalytic reduction, resulting in a decreased cathode peak. However, more ACT^+^ was consumed by HMF oxidation, and no excess ACT^+^ was used for electrocatalytic reduction, resulting in the disappearance of the cathode peak.

In order to demonstrate the electrocatalytic oxidation ability of HMF by ACT, the HMF electrolyte with or without the ACT catalyst was electrolyzed at 2.10 V vs. RHE (1.30 V vs. SCE). When the electrolyte did not contain the ACT catalyst, the HMF conversion was only 8.07% after reaching the theoretical conversion charge (174.00 C), and the FDCA yield and faradaic efficiency were less than 1.00%. When 10.00 mM ACT was added to the electrolyte, HMF conversion markedly increased to 97.20%, and the FDCA yield and faradaic efficiency memorably increased to 96.91% and 96.62%, respectively. This result indicated that the ACT could effectively electrocatalytically oxidize HMF to FDCA, while the CC electrode had little catalytic ability for HMF (Figure 5c) [27].

Additionally, the ACT-mediated electrocatalytic oxidation of HMF at different potentials was further explored. The HMF conversion was maintained above 97.00%, and the yield of FDCA and the faradaic efficiency were both maintained above 96.00% in the potential range of 1.40~2.20 V vs. RHE (Figure 5d). The catalytic effect of ACT on HMF was not affected by the applied potentials, since no obvious correlation between them was found.

**Figure 6:**
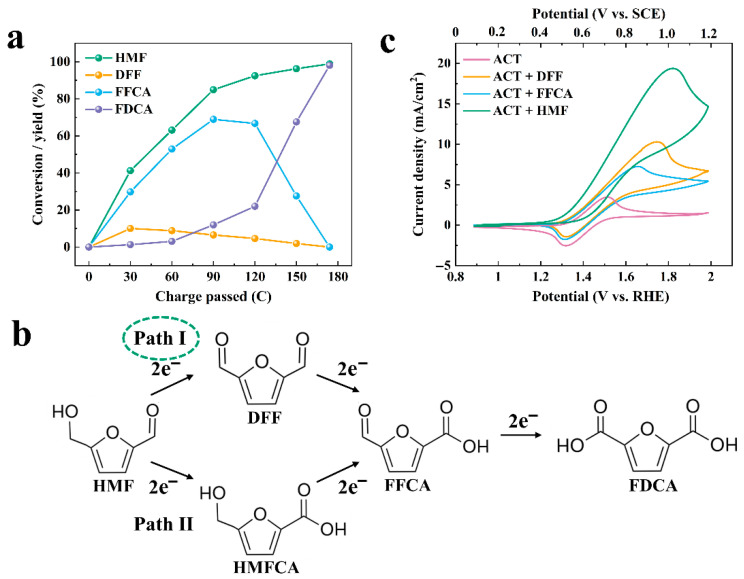
(**a**) Conversion of HMF and yield of oxidation products; (**b**) Reaction pathways for HMF oxidation to FDCA during the electrochemical oxidation of HMF; (**c**) CV curves of HMF and intermediates by ACT.

HPLC was utilized to keep track of the substrate and product concentration changes during ACT-mediated HMF oxidation. As shown in Figure 6a, during the early stage of electrolysis, the concentration of HMF gradually decreased. The concentration of the intermediate product DFF increased slightly and then decreased, while the concentration of the oxidation product FFCA continued to increase. Until the late stages of electrolysis, FFCA reached a certain concentration and was further oxidized to the target product, FDCA. Meanwhile, the intermediate product HMFCA was not detected, suggesting that FDCA was generated from ACT-mediated electrocatalytic oxidation of HMF through pathway I (Figure 6b). This pathway is different from that of our previous study, which employs heterogeneous catalyst NiMoO_4_-CNTs-CF to catalyze the oxidation of HMF to produce FDCA (Pathway II) [10].

To compare the catalytic oxidation kinetics of ACT^+^ for HMF and its intermediates, CV tests were performed in ACT solutions containing DFF, HMF, and FFCA, respectively. Since the oxidation rate of ACT^+^ to the above three substances is equal to the regeneration rate of ACT, when the oxidation kinetics were the fastest, the anode peak of ACT was enhanced most significantly, and the cathode peak of ACT^+^ was reduced most obviously. As displayed in Figure 6c, the anode peak of the ACT solution containing HMF was the highest, followed by that of DFF and FFCA, indicating that the oxidation kinetics of HMF was the fastest by ACT^+^, followed by DFF and FFCA. This result is also consistent with the conversion of HMF and the yields of FFCA and DFF, as indicated in Figure 6a.

**Figure 7:**
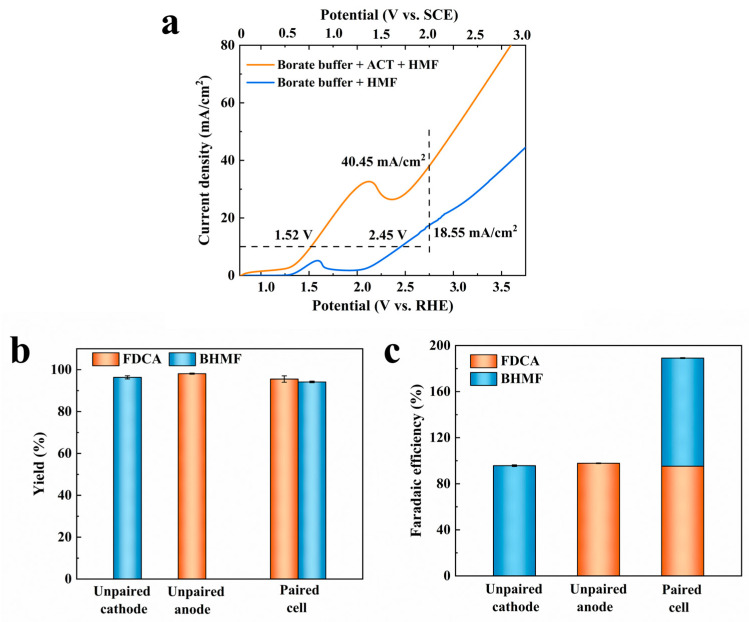
(**a**) LSV curves of HMF paired electrolysis reactions; (**b**) Yield and (**c**) faradaic efficiency of FDCA and BHMF by unpaired and paired electrolysis of HMF.

On the basis of confirming that ACT-mediated HMF catalytic oxidation can be performed, the LSV experiments were conducted to verify the possibility of the construction of HMF paired electrolysis. When HMF was added, the potentials at 10.00 mA/cm^2^ decreased from 2.45 V vs. RHE to 1.52 V vs. RHE, and the current density at 2.80 V vs. RHE increased from 18.55 mA/cm^2^ to 40.45 mA/cm^2^. This finding suggested that the paired electrochemical conversion of HMF occurred more favorably than the water electrolysis reaction (Figure 7a).

In the paired electrosynthesis system of HMF, six electrons were involved in the process of oxidizing HMF to FDCA, while two electrons were required for the hydrogenation of HMF to BHMF. To ensure the electron equilibrium between the anode and the cathode chambers, the concentration of HMF in the anode chamber was 10.00 mM, and the addition of HMF in the cathode chamber was 30.00 mM. The electrolysis was conducted at a constant potential of −0.51 V vs. RHE (−1.30 V vs. SCE). The cathode and anode reaction formulas, as well as total reaction formulas, are as follows.

Anode: HMF + 6OH^−^ → FDCA + 4H_2_O + 6e^−^

Cathode: HMF + 2e^−^ + 2H_2_O → BHMF + 2OH^−^

Overall: 4HMF + 2H_2_O → 3BHMF + FDCA

It takes 174.00 C to convert the substrate of both cells into the target products. Yields of 95.54% for FDCA and 94.14% for BHMF were obtained, which were slightly lower than those in unpaired cells (FDCA-98.10%, BHMF-96.35%) (Figure 7b). The total faradaic efficiency of 189.11% for FDCA and BHMF in the paired electrolysis system was achieved, nearly double that in unpaired cells (Figure 7c). It is indicated that the HMF electro-oxidation and electro-hydrogenation reactions could occur in pairs, and no other adverse reactions appeared.

### 2.4. Durability of Paired Electrolysis System

**Figure 8:**
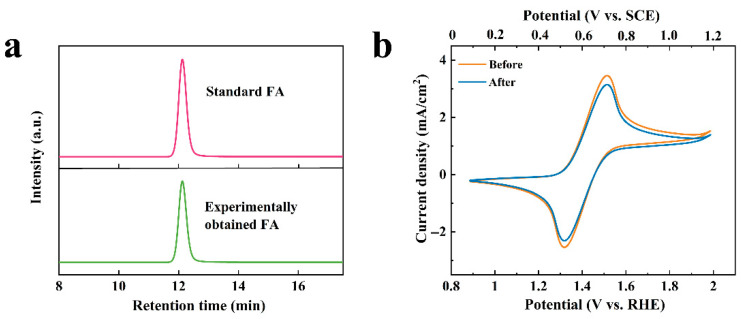
(**a**) HPLC analysis of solid precipitation from electrolyte and FA standard sample; (**b**) Comparison of CV curves of ACT solution before and after use.

To investigate the repeated stability of the paired electrolysis system, the stability of the ACT-mediated catalyzed oxidation system was explored above all. When the electrolyte contained the ACT and FUR products, it was acidified to pH 1.0 at the end of electrolysis, and the precipitated solid was compared with the FA standard product by HPLC. As displayed in Figure 8a, the standard FA and the obtained solid had the same retention time, suggesting that the solid product by the acidified separation was the FUR oxidation product FA. In addition, CV tests were performed on the used ACT and the newly prepared ACT solutions. The results showed that the CV curves of the ACT solution before and after use almost overlapped, exhibiting the same electrochemical activity (Figure 8b). The CV curve of the used ACT solution was slightly lower than that of the newly prepared ACT solution, possibly due to dilution of the ACT solution during the pH adjustment process. Moreover, the addition of substrate can be used as an anode electrolyte again after its pH is adjusted back to 9.2.

**Figure 9:**
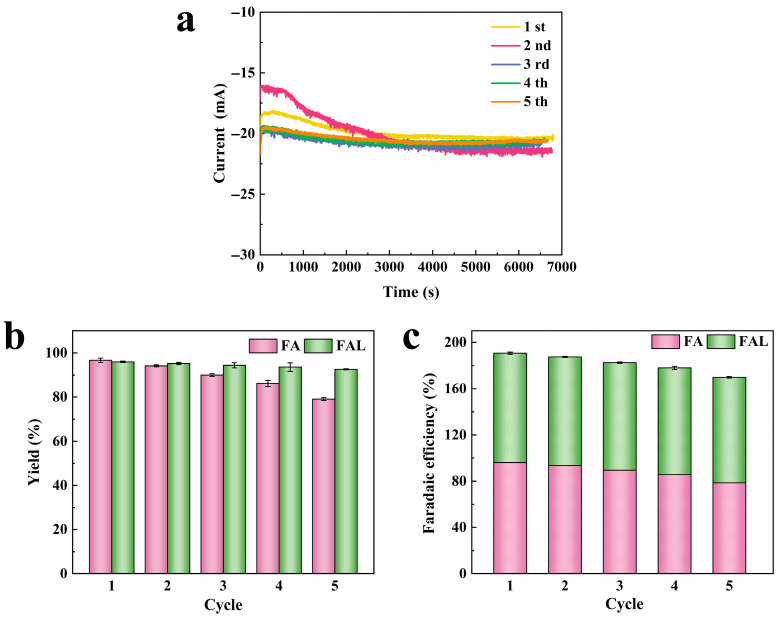
(**a**) The current-time curves of five consecutive FUR paired electrolysis; (**b**) Yield and (**c**) faradaic efficiency of cathode and anode products five consecutive FUR paired electrolysis.

Based on ensuring the reusable activity of the ACT solution, the repeatability and stability of the paired electrolysis system were further investigated. The CC and Pd/Cu-CF-II electrodes were cleaned with alcohol and DI water at the end of electrolysis. The anode electrolyte was adjusted to the initial pH and re-added to the substrate for the next electrolysis cycle, while the cathode electrolyte was replaced with a new solution containing the substrate. As displayed in Figure 9a, the i–t curves were relatively stable over five paired FUR electrolytic cycles. The yield of cathode product FAL was still above 92.00%, and the faradaic efficiency was maintained at more than 91.00%. The yield of anode product FA decreased from 96.67% to 79.07%, and the faradaic efficiency decreased from 96.04% to 78.55%. This attenuation may be attributed to dilution of the ACT due to repeated pH adjustments, resulting in a reduction in its catalytic capacity. Nevertheless, the total faradaic efficiency of the paired electrolysis system remained above 169.88% after five consecutive cycles (Figure 9b,c). The repeatable stability of the HMF paired electrolysis system was also explored, and the total faradaic efficiency of the system remained above 167.64% after five consecutive cycles (Figure S4). Additionally, the reused CC electrode was characterized by SEM. Compared with the unused CC electrode, the morphology of the reused CC electrode did not change, indicating that it possessed good stability (Figure S5). These results indicated that the constructed paired electrolysis system possessed good durability and stability. Meanwhile, the high activity and good durability of the FUR and HMF paired electrolysis system make it stand out from most of the reported electrocatalysts (Table S1 [16,18,19,20,34]).

### 2.5. Solar-Driven Paired FUR Hydrogenation and Oxidation

**Figure 10:**
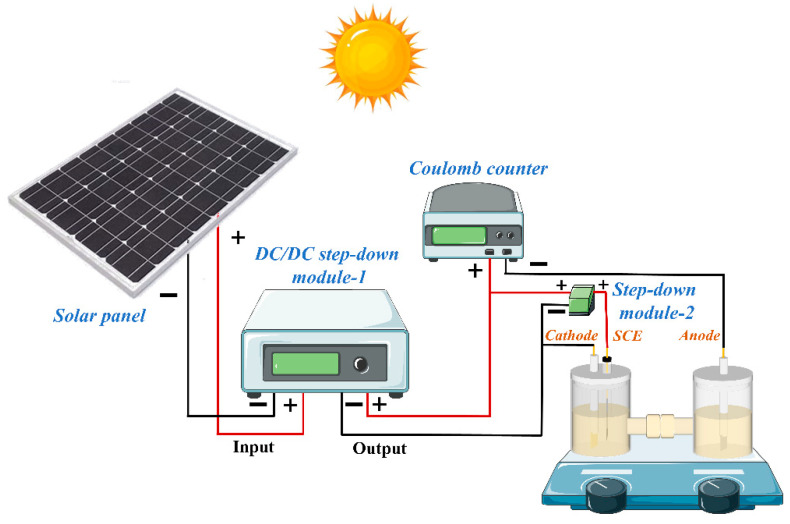
Schematic diagram of the solar-driven paired electrolysis system.

To realize a fully green process, solar energy was employed as the power source. As illustrated in Figure 10 and Figure S6, the integrated system consisted of solar panels, a DC/DC step-down module, an as-prepared coulomb counter, and the electrochemical reactor. To ensure experimental consistency and mitigate the natural light variations, an artificial Honle UV lamp was used to simulate steady solar radiation. The solar panel converted solar radiation into direct current (DC) for the paired electrolysis system. Since the output potential of the solar panel exceeded the optimal operating potential of the paired electrolysis system, a DC/DC step-down module was incorporated to precisely regulate the applied potential, thereby enabling the normal operation of the entire system.

**Figure 11:**
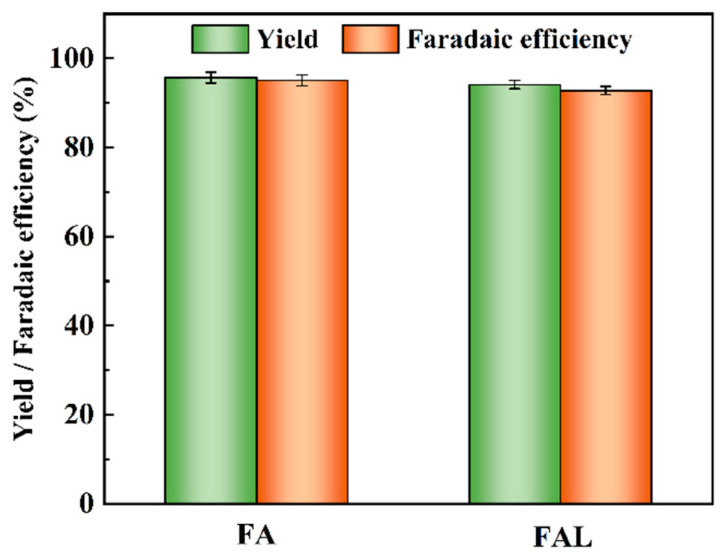
Product yield and faradaic efficiency in solar-driven paired electrolysis system.

In the configured system, the cathode potential was maintained at −1.30 V vs. RHE using a step-down module. This solar-driven setup achieved remarkable product yields of 95.71% for FA and 94.10% for FAL, with a total faradaic efficiency of 187.89% (Figure 11). The overall energy conversion efficiency from solar to chemical energy was calculated to be 24.80%, based on the photovoltaic efficiency of the monocrystalline silicon panel (15.00%) and the conversion efficiency of the DC/DC step-down module (88.00%). This result validated the high efficacy of the integrated system in achieving efficient green conversion.

## 3. Materials and Methods

### 3.1. Materials

The carbon fiber paper (CFP) was purchased from Toray Industries, Inc., Tokyo, Japan. The carbon cloth (CC) was purchased from Hong Kong Phychem Co., Ltd., Suzhou, Jiangsu, China. Nitric acid (HNO_3_) and sulfuric acid (H_2_SO_4_) were obtained from Beijing Chemical Works, Beijing, China. Furoic acid (FA, 98%), 2,5-diformylfuran (DFF, 98%), furfural (FUR, 99%), 5-hydroxymethyl-2-furancarboxylic acid (HMFCA, 98%), 5-hydroxymethylfurfural (HMF, 99%), furfuryl alcohol (FAL, 98%), 5-formylfuran-2-carboxylic acid (FFCA, 98%), 2,5-bis(hydroxymethyl)furan (BHMF, 98%), 4-acetamido-TEMPO (ACT), and 2,5-furandicarboxylic acid (FDCA, 98%) were acquired from Shanghai Macklin Reagent Co., Ltd., Shanghai, China. All reagents used were analytical grade, and deionized water was employed throughout the experiments. The DC/DC step-down module (SK80H) was purchased from Duhui Mingwu Electronics Co., Ltd., Shenzhen, Guangdong, China. The Honle UV lamp was purchased from Philips Electronics Ltd. (Mississauga, ON, Canada).

### 3.2. Electrochemical Measurements

The electrochemical analyses were conducted in a CHI 660E electrochemical workstation (CH Instruments, Inc., Shanghai, China) with a conventional three-electrode setup. The saturated calomel electrode (SCE, E _vs_. _RHE_ = E _vs_. _SCE_ + 0.244 + 0.059 × pH) worked as the reference electrode, while the CFP/CC electrode served as the counter electrode. The as-prepared Pd/Cu-CF-II electrode (1.00 cm^2^) was used as the working electrode, and the preparation method was determined by the previous study [33]. The 0.5 M borate buffer is mildly basic (pH = 9.2), which has good solubility and selectivity for the substrate as electrolyte, and can prevent side reactions or catalyst deactivation [35]. Linear sweep voltammetry (LSV) tests were carried out in 0.5 M borate buffer, with or without FUR and ACT, at a scan rate of 5.00 mV s^−1^, with iR correction. Cyclic voltammetry (CV) was performed in 0.5 M borate buffer at scan rates ranging from 20.00 to 120.00 mV/s to determine the electrochemical double-layer capacitance, which is in proportion to the electrochemically active surface area (ECSA) of CFP/CC electrode [36].

### 3.3. ACT-Mediated FUR Substrate Oxidation

A traditional H-cell setup was used, with the pre-treated Nafion 117 proton-exchange membrane dividing it into a cathode chamber and an anode chamber. The cathode electrolyte comprised 0.5 M borate buffer, and the anode electrolyte included 0.5 M borate buffer with 20.00 mM FUR and 10.00 mM ACT. The electrolyte in the cell was continuously stirred using magnetic stirrers during the reaction. Prior to testing, the solution was flushed with N_2_ to eliminate dissolved O_2_. The N_2_ flow was maintained throughout the tests.

### 3.4. Paired Electrolysis System

The paired electrolysis test was also carried out in an H-type cell system at a constant potential. The cathode electrolyte was 0.5 M borate buffer containing 20.00 mM FUR, and the anode electrolyte was 0.5 M borate buffer containing 20.00 mM FUR and 10.00 mM ACT. The electrolyte was purged with N_2_ and was stirred throughout the reaction.

Following the electrolysis procedure, the electrolyte in the anode cell was acidified to pH 1.0, facilitating the precipitation of the product in its solid form. Subsequently, the pH of the anode electrolyte was adjusted to 9.2, after which the substrate was introduced. This step was repeated five times to assess the stability of the paired electrolysis system.

### 3.5. Construction of Solar-Powered Paired Electrolysis System

The solar panel was used to replace conventional electrical energy, and step-down modules and an as-prepared coulomb counter (a tool that controls the reaction when the theoretical coulomb number is reached) were connected in series. The as-prepared coulomb counter was composed of a screen, a button, a microcontroller, and an ADC amplifier. The coulombs were obtained by the direct integration of the ADC amplifier for small currents. The assembly of the paired electrolysis system was described in Section 2.5.

### 3.6. Product Analysis

The oxidation and hydrogenation products were evaluated via HPLC (Agilent 1100, Agilent Technologies Inc., Santa Clara, CA, USA), which was furnished with two types of C18 columns (15 cm × 4.6 mm × 5 μm, Eclipse XDB-C18, Agilent Technologies, USA) (25 cm × 4.6 mm × 5 μm, Shim VP-ODS, Shimadzu Technologies Inc., Kyoto, Japan) and ultraviolet-visible detector [10,37]. The quantification and identification of each compound were based on the calibration curves and retention time derived from standard solutions.

The faradaic efficiency (FE), substrate conversion, and yield of the products (%) were determined based on the following equations.

(1) $$\mathrm{FE}\;(\%)=\frac{\mathrm{mol}\;\mathrm{of}\;\mathrm{product}\;\mathrm{formed}}{\mathrm{total}\;\mathrm{charge}\;\mathrm{passed}/(F\times n)}\times 100\%,$$

(2) $$\mathrm{Substrate}\;\mathrm{conversion}\;(\%)=\frac{\mathrm{mol}\;\mathrm{of}\;\mathrm{substrate}\;\mathrm{consumed}}{\mathrm{mol}\;\mathrm{of}\;\mathrm{initial}\;\mathrm{substrate}}\times 100\%,$$

(3) $$\mathrm{Yield}\;(\%)=\frac{\mathrm{mol}\;\mathrm{of}\;\mathrm{target}\;\mathrm{product}\;\mathrm{formed}}{\mathrm{mol}\;\mathrm{of}\;\mathrm{initial}\;\mathrm{substrate}}\times 100\%,$$

### 3.7. Energy Conversion Efficiency

The energy conversion efficiency (%) was calculated using the following equation.

(4) Energy conversion efficiency (%)=FE×CE1×CE2×100%

CE1—Photoelectric conversion efficiency (15.00%, taken from the product manual). CE2—DC/DC module energy conversion efficiency (88.00%, taken from the product manual).

### 3.8. Statistical Analysis

All analyses were performed in triplicate, and results are presented as means ± standard deviations.

## 4. Conclusions

The paired electrolysis system for biomass-derived furanal compounds was successfully constructed by the mediation of the homogeneous catalyst ACT. The substrate was oxidized by ACT^+^, exhibiting characteristics of a non-electrochemical reaction, and the catalytic activity of ACT was independent of the anode potential within the specified potential range. This property effectively addressed the challenge of the compatibility between anode and cathode potentials. The yields of anode product FA and cathode product FAL were 96.67% and 95.96%, respectively, and the total faradaic efficiency was 190.69% in the FUR paired electrolysis system. The yields of the anode product FDCA and the cathode product BHMF were 95.54% and 94.14%, respectively, and the total faradaic efficiency was 189.11% in the HMF paired electrolysis system. The total faradaic efficiency of the FUR and HMF paired electrolysis system remained above 167.64% after five consecutive electrolysis cycles, suggesting that the system possessed excellent durability. In addition, the solar-driven paired electrolysis system achieved green and high-efficiency substrate conversion, and the total faradaic efficiency was 187.89%. The paired electrolysis system has an outstanding practical pathway and economic value because it can produce two products in a single reaction and enable the reuse of ACT and electrolytes. The system provides an efficient and sustainable method for the utilization of agroforestry biomass.

## Data Availability

The original contributions presented in this study are included in the article. Further inquiries can be directed to the corresponding authors.

## Supplementary Materials

The following supporting information can be downloaded at: https://www.mdpi.com/article/10.3390/molecules31040678/s1; Figure S1: The SEM images of (a,b) CFP and (c,d) CC at different magnifications; Figure S2: Cyclic voltammetry scans over (a) CFP and (b) CC, (c) Double-layer capacitances-scan rate curves, (d) LSV curves of CFP and CC electrodes; Figure S3: FUR conversion, FAL yield and faradaic efficiency of the electrocatalytic reduction of FUR on the Pd/Cu-CF-II electrodes under different potentials; Figure S4: (a) The current-time curves of five consecutive HMF paired electrolysis, (b) Yield and (c) faradaic efficiency of cathode and anode products five consecutive HMF paired electrolysis; Figure S5: SEM of CC electrode at different magnifications after five consecutive paired electrolysis; Table S1 Comparison of the performance of FUR and HMF paired electrolysis system with other reported electrolysis system; Figure S6: The device of solar-driven paired electrolysis system.
