# Supplementary material for: Solar-Driven Paired Electrolysis System: A Green Electrosynthesis Strategy for Valorizing Agroforestry Biomass Derived Furanal Compounds
Source: Molecules. 2026 Feb 15;31(4):678. doi: 10.3390/molecules31040678 (PMC12943311; doi:10.3390/molecules31040678)
Supplement: Supplementary file 1 [file molecules-31-00678-s001.zip › molecules-4120718-supplementary.pdf]

Supplementary Material

Solar-driven paired electrolysis system: A green electrosynthesis strategy for valorizing agroforestry biomass derived furanal compounds

Yi Wu [1,2], Run Xu [3], Bowei Wang [4], Changxia Sun [2,*], Xueyong Ren [4,*], Qiang Li [2,*]

1. College of food science, Shanxi Normal University, Shanxi 030031, China

2. College of science, Beijing Forestry University, Beijing 100083, China

3. Sinopec research institute of petroleum processing Co., LTD, Beijing 100083, China

4. College of material science and technology, Beijing Forestry University, Beijing 100083, China

*Correspondence: liqiang@bjfu.edu.cn

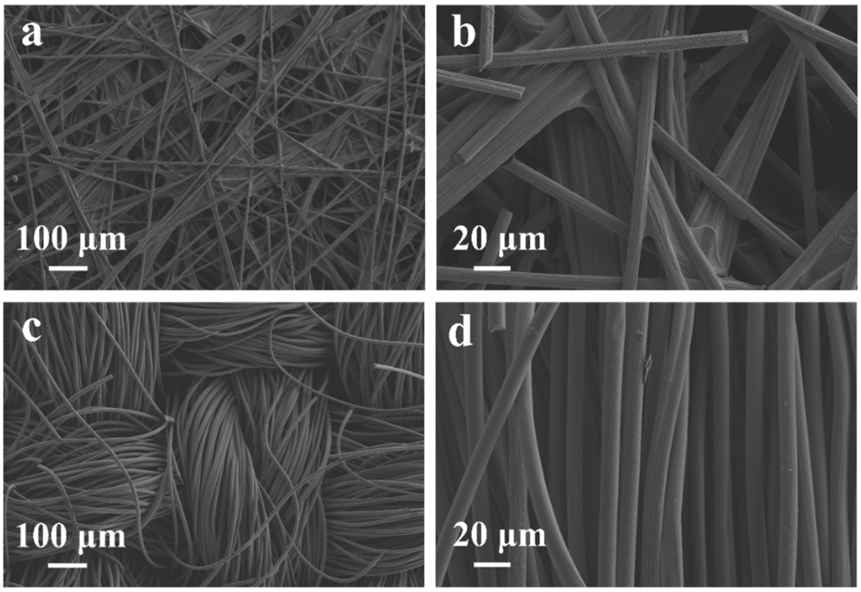

Figure S1 The SEM images of (a-b) CFP and (c-d) CC at different magnifications

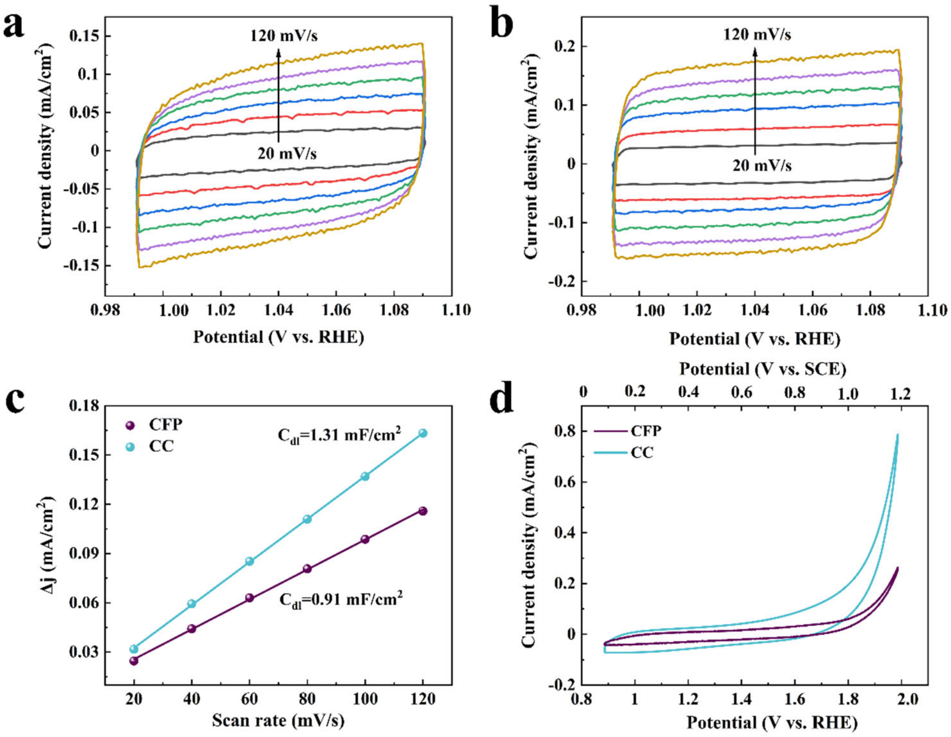

Figure S2 Cyclic voltammetry scans over (a) CFP and (b) CC; (c) Double-layer capacitances-scan rate curves; (d) LSV curves of CFP and CC electrodes

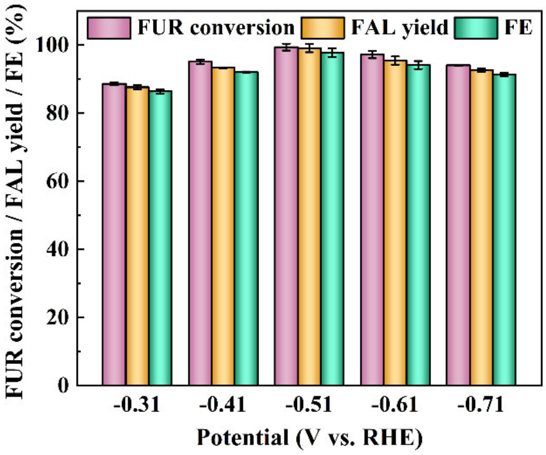

Figure S3 FUR conversion, FAL yield and faradaic efficiency of the electrocatalytic reduction of FUR on the Pd/Cu-CF-II electrodes under different potentials

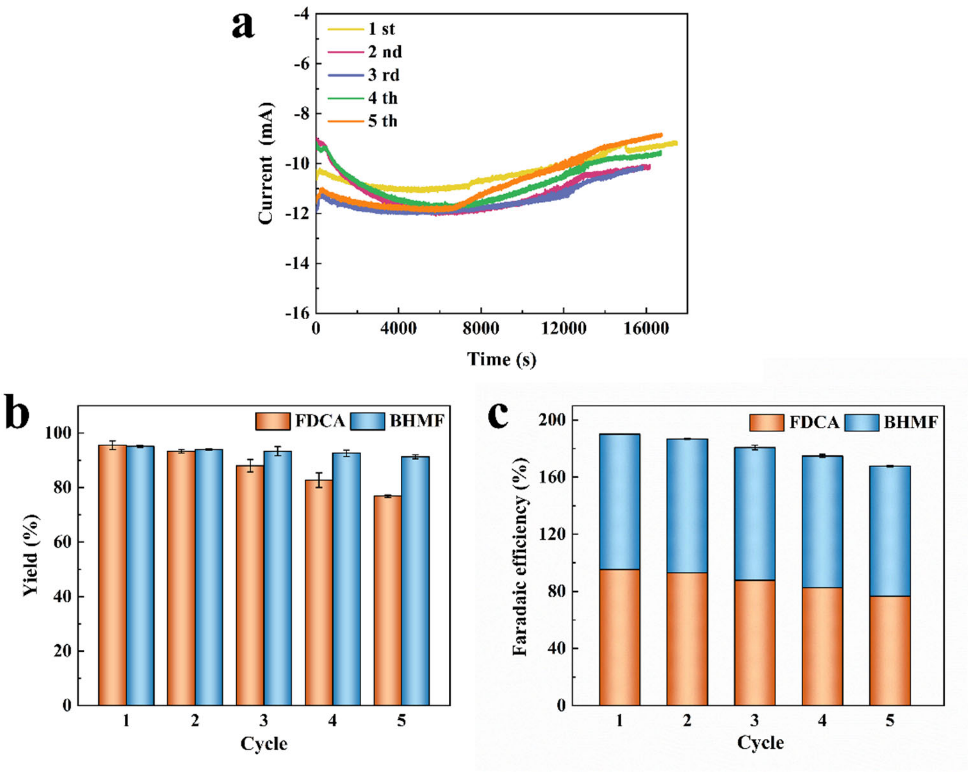

Figure S4 (a) The current-time curves of five consecutive HMF paired electrolysis; (b) Yield and (c) faradaic efficiency of cathode and anode products five consecutive HMF paired electrolysis

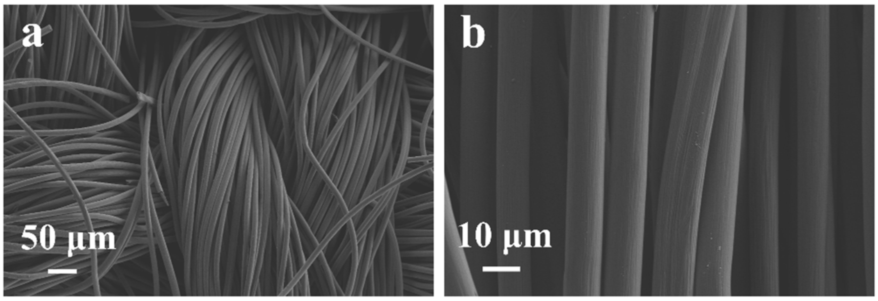

Figure S5 SEM of CC electrode at different magnifications after five consecutive paired electrolysis

Table S1 Comparison of the performance of FUR and HMF paired electrolysis system with other reported electrolysis system

**Substrate**	**Anode product**	**Cathode product**	**Total FE (%)**	**Cycles**	**Ref.**
FUR	FA	FAL	190.69	5	**This work**
HMF	FDCA	BHMF	189.11	5	**This work**
HMF	FDCA	BHMF	187	-	[1]
HMF	FDCA	BHMF	163	-	[2]
HMF	FDCA	HEMF	110 (yield)	-	[3]
HMF	FDCA	DHMTHF	170	8	[4]
4-methoxybenzyl alcohol<br>1-hexyne	4-methoxybenzaldehyde	1-hexene	140 (current efficiency)<br>-	-	[5]

References:

[1] Green Chem., 2019, 21, 6210-6219.

[2] Green Chem., 2021, 23, 5056-5063.

[3] Ind. Eng. Chem. Res., 2022, 61, 1912-1919.

[4] Adv. Funct. Mater., 2019, 29, 1904780.

[5] Nat. Catal., 2018, 1, 501-507.

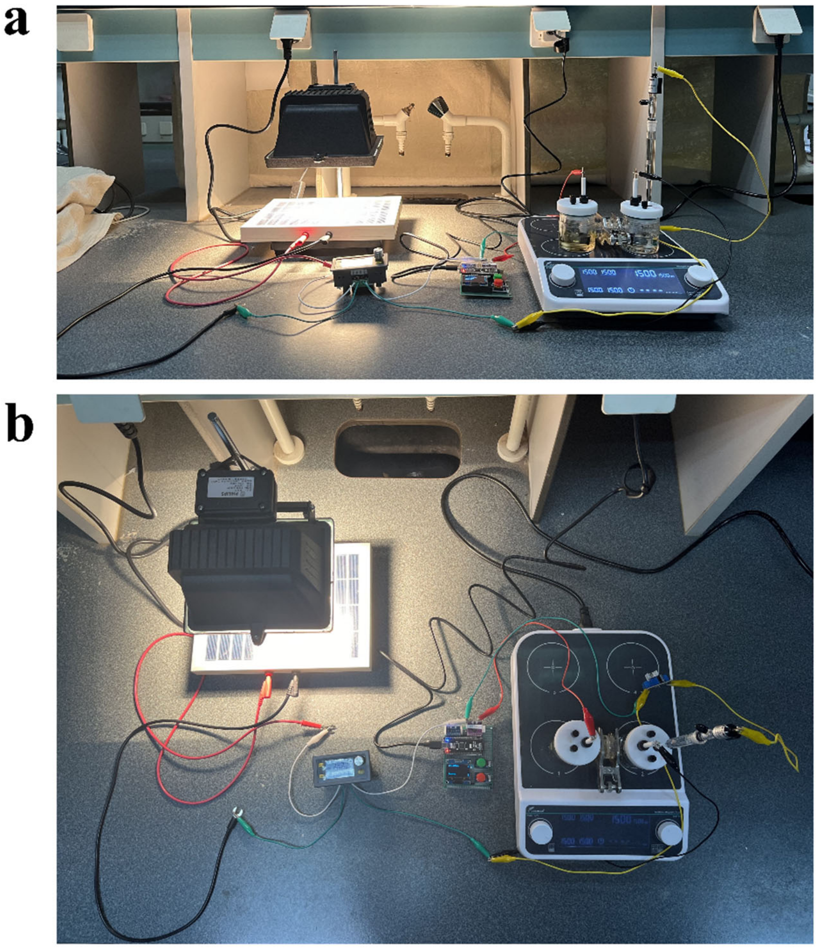

Figure S6 The device of solar-driven paired electrolysis system
